# The Adenosine A2A Receptor Agonist Accelerates Bone Healing and Adjusts Treg/Th17 Cell Balance through Interleukin 6

**DOI:** 10.1155/2020/2603873

**Published:** 2020-04-23

**Authors:** Xi Zheng, Dong Wang

**Affiliations:** ^1^Department of SICU, Beijing Chaoyang Hospital, Capital Medical University, Chaoyang District, Beijing, China; ^2^Department of Orthopedics, Beijing Chaoyang Hospital, Capital Medical University, Chaoyang District, Beijing, China

## Abstract

The aim of this study was to explore the effect of adenosine A2A receptor agonists on fracture healing and the regulation of the immunity system after bone fracture. We implanted fibrin gel containing adenosine A2A receptor agonist CGS 21680/inhibitor ZM 241385/saline locally in rat tibial fracture models, finding that the adenosine A2A receptor agonist could promote fracture healing. At the same time, the adenosine A2A receptor agonist decreased the level of IL-6 in blood and the fracture area, increased Treg cells, and decreased Th17 cells in blood of bone fracture rats. Further, tibial fracture rats implanted with the adenosine A2A receptor agonist gel were injected with IL-6. We found that IL-6 could reverse the effect of adenosine A2A receptor agonists on fracture healing and Treg/Th17 cells in blood. Through the above results, we believe that the adenosine A2A receptor agonist can promote fracture healing and regulate Treg/Th17 cells in blood of rats with fractures. These effects are related to IL-6.

## 1. Introduction

How to promote bone healing and reduce the rate of nonunion is a hot topic within long-term research [1, 2]. In 2018, the global incidence of fracture was around 2%. Although anatomical reduction and rigid fixation are performed strictly in accordance with the treatment norms, delayed union or nonunion still occurs at about 5–10% because of the complex fracture healing process [1, 3–5]. Tibial shaft fractures are the most common long bone fracture and are prone to complications such as nonunion [6]. The incidence of complications in tibial shaft fractures, such as delayed union or nonunion, is 4–48% [7–9].

Currently, there are many methods used for promoting fracture healing in clinics [1, 2, 10–12]. Surgical intervention and autologous bone transplantation are the gold standard of current treatment in the event of fracture nonunion, but the trauma is so large that some patients may need multiple surgeries for years [13–15]. Autologous bone is generally taken from the iliac crest or fibula of the patient, which is more damaging to the patient and is prone to infection after surgery. The cell components in allogeneic bones are mostly dead and do not have their own osteogenic ability. Studies have found that allogeneic bone treatment for nonunion fractures takes about 12 months for allogeneic bone surface union; indeed, internal osteogenesis is very slow, occurring at a rate of only 15–20% within five years, and deep repair hardly occurs [13, 14].

Bone morphogenetic protein-2 (BMP-2) has been studied to fracture nonunion [15, 16]. Although BMP-2 has an obvious effect in promoting fracture healing, ectopic ossification can easily occur. Croes et al. [17] supported that BMP-2 can induce ectopic bone formation and enhance interleukin 17 production. Carragee et al. [18] analyzed the safety and complications of BMP-2 on spinal fusion patients. They found that anterior cervical fusion with rhBMP-2 has an estimated 40% greater risk of adverse events in the early postoperative period, including life-threatening events. Indeed, many studies have found the negative effects of BMP-2 [2, 17–19].

Recently, some studies have proposed that the adenosine A2A receptor agonist can promote fracture healing with an efficacy similar to BMP-2 [20]. At present, animal experiments have not found any adverse reactions of the A2A agonist, such as infection and heterotopic ossification. Studies have reported that the A2A receptor agonist could regulate blood Treg/Th17 cells to regulate immune reaction in an asthma model [21]. Indeed, immune regulation is closely related to fracture healing. Treg cells participate in fracture healing mainly through three mechanisms: (1) expressing cytotoxic T lymphocyte antigen-4 (CTLA-4) on the surface of cells directly contacting with target cells to inhibit the damage of inflammatory cells to bone tissue, (2) promoting osteoblast activity while inhibiting osteoclast activity, and (3) producing cytokines such as TGF-beta and IL-10 [21–23]. Activated Th17 cells regulate fracture healing mainly through the following three ways: (1) enhancing the expression of IL-17 in the fracture site, (2) enhancing the activity of osteoclasts, and (3) blocking the formation of osteoblasts and inhibiting the activity of osteoblasts [21–23]. We speculate that the A2A receptor agonist can regulate the above cell balance in a rat fracture model. IL-6 is one important regulator of blood Treg/Th17 cell balance [24]. Currently, the A2A receptor agonist can regulate IL-6 in other diseases [25, 26]. We speculate that the A2A receptor agonist regulates the above cell balance after fracture through IL-6.

## 2. Material and Methods

### 2.1. Animals

Female adult Sprague-Dawley (SD) rats (body weight: 335.27 ± 21.16 g, Charles River Laboratories, Beijing) were used under a protocol approved by the Capital Medical University Committee on the Use of Animals in Research and Education. In addition, all procedures complied with the Animal Research: Reporting of In Vivo Experiments (ARRIVE) guidelines and carried out in accordance with the National Institutes of Health guidelines for the care and use of laboratory animals. The rats were kept in separate cages with a controlled condition of 12 h light/12 h dark at 23.5°C, humidified at 35%, and fed a sterilized chow diet with free water intake. All the experiments used 3% isoflurane inhalation anesthesia with a 2 l/min oxygen flow rate. At the end of the experiments, the rats were euthanized by excessive blood loss under inhalation anesthesia.

### 2.2. Preparation of the Fibrin Glue Drug Delivery System

The preparation of the fibrin glue and the selection of the A2A receptor agonist and antagonist doses were based on the methods reported by several articles [27–30]. Briefly, fibrinogen powder (Solarbio, China) was dissolved in physiological saline into a concentration with 10 mg/ml at 37°C. Thrombin powder (Solarbio, China) was dissolved in 40 mmol/l calcium chloride solution, and the concentration was 20 *μ*g/ml. Then, 5 mg CGS 21680 (specific adenosine A2A receptor agonist; Selleck, USA) or ZM 241385 (specific adenosine A2A receptor antagonist; Selleck, USA) was dissolved in 75 *μ*l DMSO and mixed with thrombin solution into a concentration with 16 mg/ml CGS 21680 or ZM 241385 thrombin solution. Afterwards, 125 *μ*l CGS 21680, ZM 241385, or thrombin solution (with DMSO as the solvent accelerating agent) was mixed with 125 *μ*l fibrinogen solution to form a fibrin gel drug delivery system after 1 min of mixing.

### 2.3. The Making of the Fracture Model

**Figure 1:**
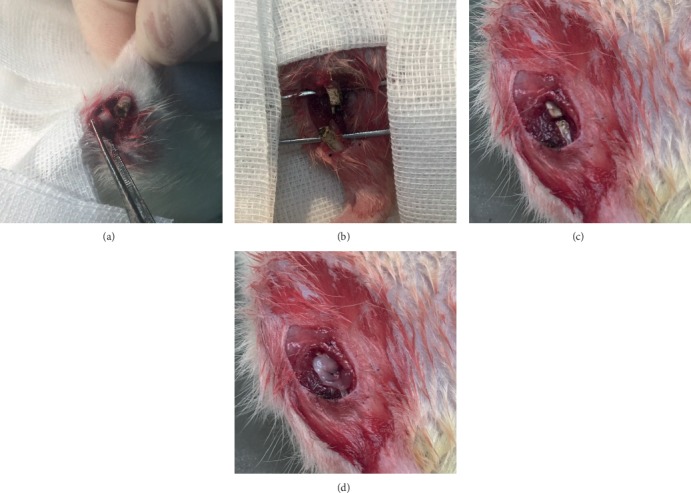
Rat fracture model and fibrin gel implantation. (a) A fracture of the middle tibial shaft on the right side of the rat was made. (b) The periosteum and the medullary cavity in the area of 5 mm above and below the fracture end were burned. (c) A 0.8 mm Kirschner wire was used to fix the fracture, and a gap of 1 mm was left at the fracture end. (d) Local fibrin gel placement in the fracture.

The fracture model was similar to previous literature reports [31–33]. Briefly, anesthesia was performed by using 3% isoflurane with an oxygen flow rate of 3 l/min. After successful anesthesia, the rats were supine on the animal operating table. The right lower limb was shaved, disinfected by 75% ethanol three times, and paved with a sterile sheet. The medial incision of the right lower limb was taken, and the length was 5 mm. The skin and subcutaneous tissue were cut to expose the middle tibia. The muscle around the middle tibia was dissected from the anterior to the bilateral, and a bone saw was used to make a transverse fracture of the middle tibia. An electric knife was used to burn the upper and lower 5 mm periosteum of the fracture line. A 0.8 mm Kirschner wire (Zimmer, USA) was retrograde inserted into the proximal tibial bone marrow cavity using a hand drill. After penetrating the skin, the Kirschner wire was inserted into the distal tibial bone marrow cavity to fix the fracture. There was a 1 mm gap at the fracture site. The muscles and skin were sutured with a 5-0 absorbable band (Ethicon, USA). Then, 80,000 IU of a penicillin intramuscular injection was performed at days 1, 2, and 3 after being modeled to prevent infection (Figure 1).

### 2.4. Animal Grouping

240 rats were divided into five groups: A2A agonist group, A2A antagonist group, IL-6+agonist group, PS+fibrin glue group, and fibrin glue group.

Fibrin gel of the A2A agonist and IL-6+agonist groups (fibrin gel containing CGS 21680), the A2A antagonist group (fibrin gel containing ZM 241385), and the PS+fibrin glue and fibrin glue groups (fibrin gel without drug) was placed at the fracture site after the fracture was fixed with the Kirschner wire. The IL-6+agonist group or PS+fibrin glue group received 1 mg/kg IL-6 or physiological saline by tail vein injection at days (after being modeled) 1, 4, 7, 10, and 13. The rats of all groups were sacrificed at days (after being modeled, 8 rats at each date) 7, 14, 28, 42, 56, and 84.

### 2.5. The Evaluation of Fracture Healing

#### 2.5.1. X-Rays

An in vivo multispectral FXPRO imaging system (Bruker, Germany) was used to take X-rays. The exposure time was set at 30 seconds, and the field of vision (FOV) was set at 120 mm. The pixel size of the obtained images was adjusted to 2000 × 2000. Adobe Photoshop CS3 was used to pick the region of interest (ROI) 5 mm above and below the fracture line. In addition, Image-Pro Plus 6.0 (IPP6.0) analyzed the grayscale value of bone and callus tissue and modified the color. Green represents callus tissue, and red represents bone tissue. Then, the ratio of the area of the callus to the bone tissue was calculated via IPP6.0.

#### 2.5.2. Micro-CT

A micro-CT machine (Bruker Skyscan 1176, Belgium) was used to take micro-CT detections. The Kirschner wire was taken out before micro-CT examination. The source voltage was set at 65 kV, current was 381 *μ*A, and filter was Al 1 mm. The rotation step was set at 0.5°, and the total rotation angle was 180°. NRecon 1.6.10.2 (Bruker, Belgium) made a two-dimensional reconstruction, and the ROI was 200 axial slices above and below the fracture line. Sequential images were obtained at slice distances of 18 *μ*m. The pixel size of the obtained image was 2000 × 2000. Mimics research 20.00 was used for three-dimensional reconstruction. The Hounsfield unit thresholding of bone was 226 to 2500, and that of callus was 50 to 226. Magics software calculated the bone volume (BV) and callus volume (CV).

#### 2.5.3. Fracture Tissue Section Staining

HE staining was used. The image processing method was the same as the X-ray image. Briefly, the pixel size of the images was 2000 × 2000. The ROI was 5 mm above and below the fracture line and was set by Adobe Photoshop CS3. The callus area was analyzed, and the ratio of the area of the callus to the bone tissue was calculated using IPP6.0.

### 2.6. The Evaluation of IL-6 Expression

#### 2.6.1. Serum IL-6

The detection time points were days (after being modeled) 7, 14, 28, 42, 56, and 84. All the processes were strictly in accordance with user instructions.

#### 2.6.2. Bone Tissue IL-6 Immunohistochemistry

The detection time points were days (after being modeled) 7, 14, 28, 42, 56, and 84. The IL-6 rabbit polyclonal antibody (Affinity Biosciences, China) (1 : 200 for immunohistochemistry) and goat anti-rabbit IgG HRP polyclonal antibody (Affinity Biosciences, China) (1 : 200 for immunohistochemistry) were used. DAB staining was used, and the pixel size of the images was 2000 × 2000. The ROI was 5 mm above and below the fracture line, which was determined using Adobe Photoshop CS3. The brown area was analyzed, and the ratio of the brown area to the bone tissue was calculated by IPP6.0.

### 2.7. The Evaluation of the Treg/Th17 Ratio of Blood

The detection time points were days (after being modeled) 7, 14, 28, 42, 56, and 84. The details of the test were as follows. Briefly, rat blood was collected and placed in a 15 ml centrifugal tube containing a rat lymphocyte isolate solution (Servicebio, China), and this was centrifuged at 1800 rpm/min for 25 min. BMC was retained between the plasma layer and the isolate solution layer and extracted to be mixed with a DPBS solution (Biyuntian, China), then centrifuged at 1500 rpm/min for 10 min. Precipitation was resuspended with 5 ml RPMI-1640 cell complete medium (10% fetal bovine serum, 1% penicillin streptomycin RPMI-1640 medium, Sigma, USA) and cultured for 12 hours. A 2 *μ*l/ml cell stimulation cocktail (500x) (eBioscience, USA) was used to stimulate the cells. The FITC-CD4 monoclonal antibody (OX35) (1 : 200; eBioscience, USA) and PerCP-Cyanine5.5-IL-17A monoclonal antibody (eBio17B7) (1 : 200; eBioscience, USA) were used to calculate the ratio of Th17. The cells were incubated with the CD4 monoclonal antibody at 4°C for 30 min. A transcription staining buffer kit (eBioscience, USA) was used to immobilize the cells and break the cell membranes. All the processes were strictly in accordance with the instructions. A flow cytometry perm buffer (eBioscience, USA) was used to wash and dilute the IL-17A monoclonal antibody. The cells were also incubated with the IL-17A monoclonal antibody at 4°C for 30 min. Then, FCM (BD FACSCanto II, USA) was used to test the ratio of Th17. The FITC-CD4 monoclonal antibody (OX35) (1 : 200; eBioscience, USA), PE-CD25 monoclonal antibody (OX39) (1 : 100; eBioscience, USA), and APC-FOXP3 monoclonal antibody (FJK-16s) (1 : 20; eBioscience, USA) were used to calculate the ratio of Treg. The cells were incubated with a CD4 monoclonal antibody and CD25 monoclonal antibody at 4°C for 30 min. A transcription staining buffer kit was used to immobilize the cells and break the cell membranes. A flow cytometry perm buffer was used to wash and dilute the FOXP3 monoclonal antibody. The cells were also incubated with a FOXP3 monoclonal antibody at 4°C for 30 min. Then, FCM (BD FACSCanto II, USA) was used to test the ratio of Treg.

### 2.8. Statistical Analysis

SPSS 24.0 was used to analyze the data. Measurement data were reported as mean ± deviation, and enumeration data were reported as percentages. If the measurement data displayed variance homogeneity, repeated measurement data were analyzed by a variance analysis of repeated measurement. A one-way ANOVA was used to compare the differences among the groups. The least significant difference (LSD) tests were used for multiple comparisons. If the measure data did not display variance homogeneity, the Mancini *U* test was used to compare the differences among the groups. The Dunnett T3 test was used for multiple comparisons. A chi-squared test was used to compare the enumeration data among the groups. *p* value < 0.05 was accepted as statistically significant.

## 3. Results

### 3.1. The Adenosine A2A Receptor Agonist Accelerates Fracture Healing

**Figure 2:**
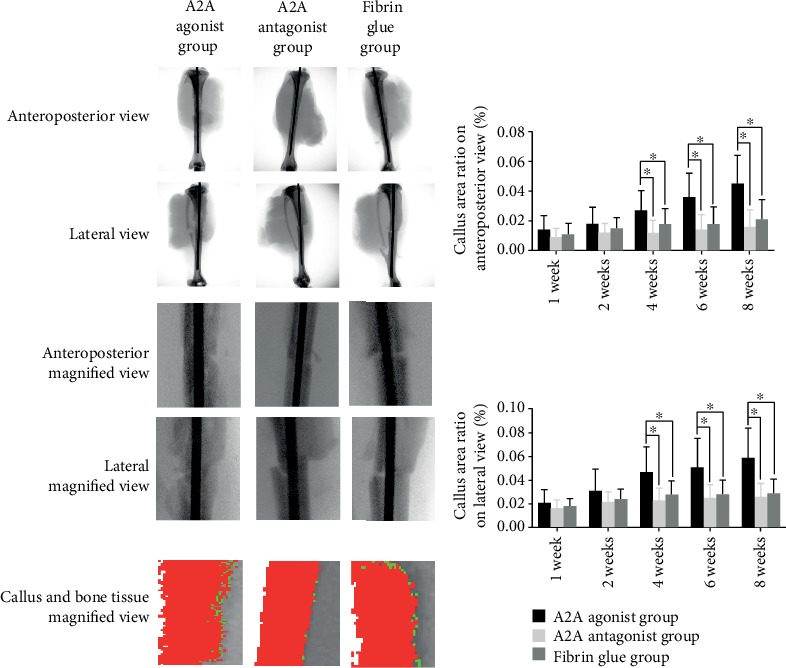
X-ray results of the effect of the adenosine A2A receptor agonist on fracture healing. The X-rays were taken in the front and rear position and lateral position. The image of 5 mm above and below the fracture line was captured using Photoshop software. The images were imported into IPP software for analysis. Red represents bone mineralized tissue, and green represents callus tissue. The area of callus tissue in the anterior and posterior and lateral positions was calculated as callus tissue + bone mineralized tissue area. The results show that the adenosine A2A receptor agonist could significantly increase the callus tissue area (callus tissue + bone mineralized tissue area) on the *X* line at 4, 6, and 8 weeks after modeling in rats, and the difference was statistically si gnificant (^∗^*p* < 0.05).

**Figure 3:**
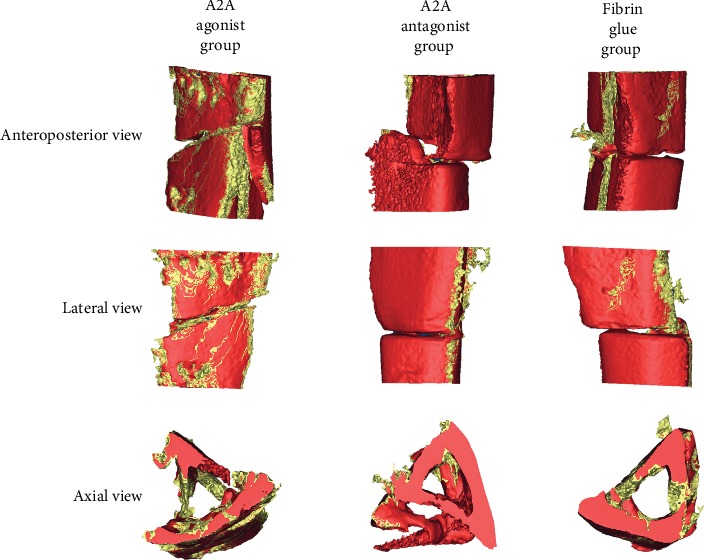
CT image of the adenosine A2A receptor agonist on promoting fracture healing. A CT scan was performed on 200 layers above and below the fracture line. The data were imported into NRecon 1.6.10.2 for two-dimensional reconstruction. After reconstruction, the data were put into Mimics research 20.00 for 3D reconstruction. The bone mineralized tissues were marked with red and callus tissues with yellow. Callus formation was observed from the anteroposterior, lateral, and axial positions. The adenosine A2A receptor agonist is shown to promote callus formation.

**Figure 4:**
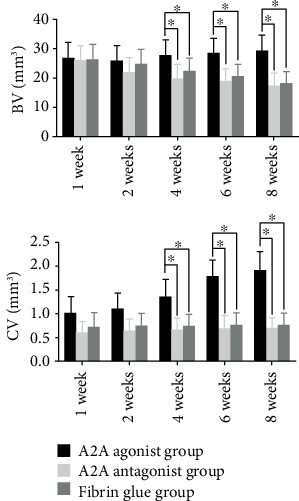
CT results of the adenosine A2A receptor agonist promoting fracture healing. We compared the volume of bone mineralized tissue and callus tissue after 3D reconstruction. The adenosine A2A receptor agonist was found to increase the volume of bone mineralized tissues and callus tissues at weeks 4, 6, and 8 in bone fracture rats, and the difference was statistically significant (^∗^*p* < 0.05).

It was found that the A2A receptor agonist could increase the callus/bone tissue values in the 5 mm area above and below the fracture line of rats at 4, 6, and 8 weeks with a statistical difference. However, there was no significant difference in the callus/bone tissue values at 1, 2, 4, 6, and 8 weeks between the adenosine A2A receptor inhibitors and the control group. There was no significant difference in the callus volume and bone mineralized tissue volume between the antagonist group and the control group at 1, 2, 4, 6, and 8 weeks (Figures 2–4).

### 3.2. The Adenosine A2A Receptor Agonist Reduced the Serum and Bone Fracture Tissue IL-6 Level

**Figure 5:**
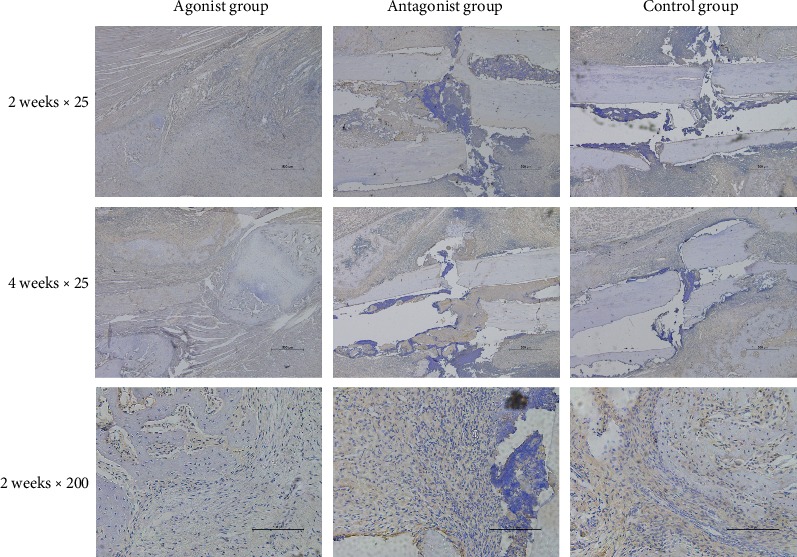
Results of IL-6 immunohistochemistry. The results showed that the adenosine A2A receptor agonist could reduce the expression of IL-6 in the fracture area, while the adenosine A2A receptor inhibitor could increase the expression of IL-6.

**Figure 6:**
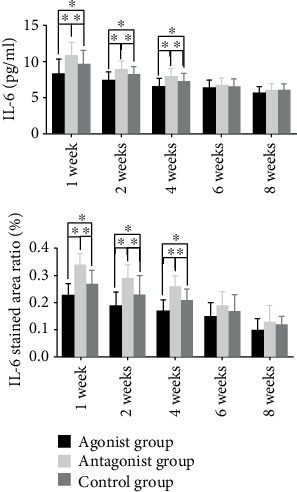
Effects of the adenosine A2A receptor agonist on IL-6 in blood and IL-6 in fracture rats. It was found that the adenosine A2A receptor agonist could reduce the level of IL-6 in the blood and local tissues of rats at 1, 2, and 4 weeks after modeling, while the adenosine A2A receptor inhibitor could increase the level of IL-6 in the blood and local tissues of rats at 1, 2, and 4 weeks after modeling. The difference was statistically significant (^∗^*p* < 0.05).

It was found that adenosine A2A receptor agonists could reduce the level of IL-6 in the serum and local fracture tissues of rats at 1, 2, and 4 weeks, and the difference was statistically significant. The adenosine A2A receptor inhibitor can increase the level of IL-6 in the serum and local fracture tissues of rats at 1, 2, and 4 weeks; the difference was found to be statistically significant (Figures 5 and 6).

### 3.3. The Adenosine A2A Receptor Agonist Increased the Ratio of Treg Cells and Decreased the Ratio of Th17 Cells in Rat Blood

**Figure 7:**
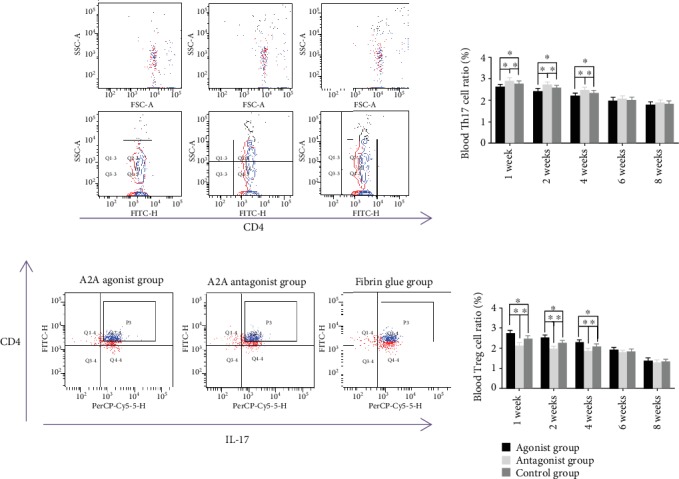
Effects of the adenosine A2A receptor agonist on Treg cells and Th17 cells in the blood of fracture rats. Flow cytometry showed that the adenosine A2A receptor agonist could reduce the proportion of Th17 cells in the blood of all CD4+ cells and increase the proportion of Treg cells in the blood of all CD4+ cells in the blood of rats at 1, 2, and 4 weeks after modeling. The adenosine A2A receptor inhibitor can increase the proportion of Th17 cells in the blood of all CD4+ cells and decrease the proportion of Treg cells in the blood of all CD4+ cells. The difference was statistically significant (^∗^*p* < 0.05).

Flow cytometry was used to observe the proportion of the Treg and Th17 cells in CD4+ cells. It was found that adenosine A2A receptor agonists could decrease the proportion of Th17 cells in the blood of fracture rats at 1, 2, and 4 weeks and increase the proportion of Treg cells in the blood of fracture rats at 1, 2, and 4 weeks. The adenosine A2A receptor inhibitor can reduce the proportion of Treg cells in the blood of fracture rats at 1, 2, and 4 weeks and increase the proportion of Th17 cells in the blood of fracture rats at 1, 2, and 4 weeks (Figure 7).

### 3.4. IL-6 Could Reverse the Promoting Efforts of the Adenosine A2A Receptor Agonist on Bone Healing and the Efforts of the Adenosine A2A Receptor Agonist on the Ratio of Treg/Th17 Cells in Blood

**Figure 8:**
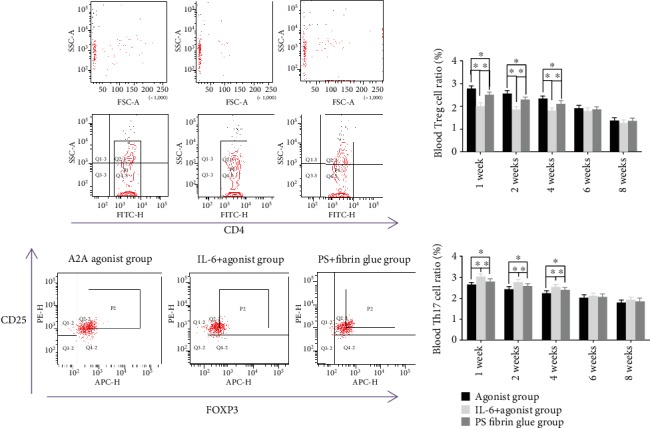
Effect of IL-6 on the adenosine A2A receptor agonist regulating the Treg and Th17 cells in the blood of bone fracture rats. It was found that IL-6 could reverse the adenosine A2A receptor agonist and increase the proportion of Treg cells and Th17 cells in all blood CD4+ cells in bone fracture rats at 1, 2, and 4 weeks. The difference was statistically significant (^∗^*p* < 0.05).

The nonunion model of SD rats was established. The fibrin gel drug delivery system was implanted into the fracture site. IL-6 was injected into the tail vein of SD rats. X-ray and uCT were used to observe bone formation in the fracture area of the rats. Flow cytometry was used to observe the proportion of Treg cells and Th17 cells in the blood of rats relative to CD4+ cells. It was found that adenosine A2A receptor agonists could increase the proportion of Treg cells in the blood of rats at 1, 2, and 4 weeks and decrease the proportion of Th17 cells in the blood at 1, 2, and 4 weeks. IL-6 reversed the effects of adenosine A2A receptor agonists on the Treg and Th17 cells in the blood of rats with a bone fracture. The proportion of Treg cells in the blood of rats treated with adenosine A2A receptor agonists and IL-6 was lower than that of the normal control group at 1, 2, and 4 weeks, and the proportion of Th17 cells in the blood of rats treated with adenosine A2A receptor agonists and IL-6 was higher than that of the control group at 1, 2, and 4 weeks. The difference was statistically significant (Figure 8).

**Figure 9:**
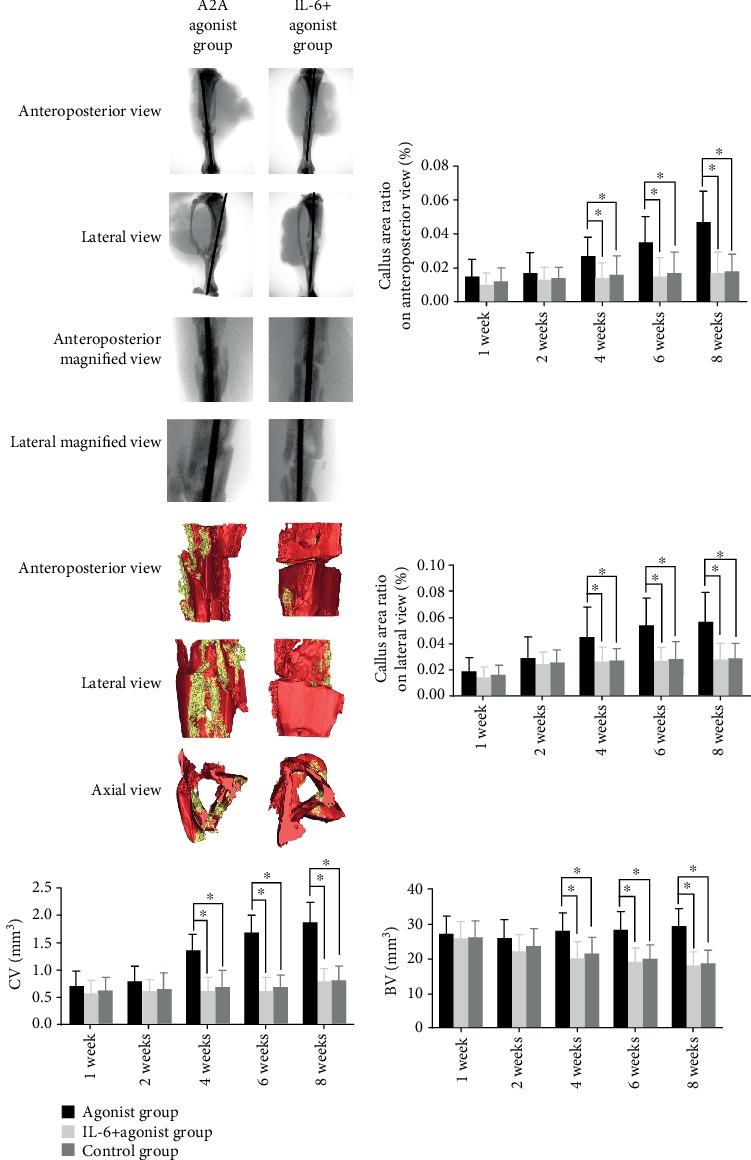
Effect of IL-6 on the adenosine A2A receptor agonist for promoting fracture healing. It was found that IL-6 reversed area on X-ray and lateral X-ray before and after 4, 6, and 8 weeks; it also reversed the volume of bone mineralized tissue and callus volume in three-dimensional CT reconstruction at 4, 6, and 8 weeks. The difference was statistically significant (^∗^*p* < 0.05).

Adenosine A2A receptor agonists also increased the callus/bone tissue (callus+bone mineralized tissue) values at 4, 6, and 8 weeks when the anterolateral tibial was observed with X-ray, and the volume of bone mineralized tissue and callus tissue at 4, 6, and 8 weeks increased when this was analyzed with uCT. The difference was statistically significant. IL-6 can reverse the effect of the adenosine A2A receptor agonist on fracture healing in rats. At 4, 6, and 8 weeks, the callus/bone tissue values of ADA receptor agonists combined with IL-6 rats were lower than those of the control group, and the volume of bone mineralized tissue and callus tissue of ADA receptor agonists combined with IL-6 rats at 4, 6, and 8 weeks was lower than that of the control group. The difference was statistically significant (Figure 9).

## 4. Discussion

Promoting fracture healing is an old topic, especially tibial shaft fracture healing [1, 7, 34, 35]. With the progress of science and technology, many methods have been found to promote fracture healing. However, with the widespread application of these techniques, there are more and more complications, such as infection, bone resorption, heterotopic ossification, allergy, and even shock, endangering the lives of patients. At present, there is no safe and reliable method to promote fracture healing.

Recently, adenosine A2A receptor agonists have been reported to promote skull repair in mice, but the mechanism is not clear [20, 36, 37]. Adenosine is an older drug mainly used in the cardiovascular field. Currently, no adverse reactions such as heterotopic ossification and infection of the local application of adenosine A2A receptor agonists have been found in animals. By studying the process of fracture healing, we know that blood supply is the foundation of fracture healing. Without a proper blood supply, the fracture will not heal. In the current study, a rat model of tibial shaft fracture was established by using the method reported in numerous studies [31–33].

In the present study, adenosine A2A receptor agonists were found to promote tibial shaft fracture healing in rats. Intravenous injection of IL-6 after modeling reversed the effect of the adenosine A2A receptor agonist on fracture healing. Here, the promotion of fracture healing by adenosine A2A receptor agonists may be related to IL-6. Adenosine A2A receptor agonists, as an immunoregulatory factor, play an important role in regulating inflammation in many diseases [21, 25, 26]. In the current study, we found that adenosine A2A receptor agonists can regulate the proportion of Treg cells and Th17 cells in the blood of bone fracture rats. Adenosine A2A receptor agonists increase Treg cells in rat blood and decrease Th17 cells. Moreover, the tail vein injection of IL-6 was found to reverse this regulatory effect. The current study preliminarily explored the effect of adenosine A2A receptor agonists on tibial shaft fracture healing and the regulation of body immunity after fracture, hence laying a foundation for further mechanism research and promotion.

The physiological role of adenosine has been recognized for more than 80 years [20, 37]. After fracture, local cells secrete adenosine, which plays a regulatory role by activating G protein-coupled receptors on cell membranes [37]. The adenosine A2A receptor is closely related to bone metabolism, and their agonists inhibit osteoclast function, reducing bone destruction in osteosarcoma patients. A lack of A2A can lead to bone loss. Adenosine A2A may be associated with methotrexate, inhibiting bone loss in patients with rheumatoid arthritis. In addition, adenosine A2A receptor agonists also play a role in bone marrow mesenchymal stem cells and osteoblasts [20, 37]. Adenosine A2A receptor agonists can also recruit bone marrow mesenchymal stem cells and activate osteoblasts [20, 37]. In addition, it has been reported that adenosine A2A receptor agonists can alleviate the damage of osteoblasts caused by hydrogen peroxide [20, 37].

Mediero et al. [36] found that a local application of adenosine A2A receptor agonists could promote bone repair as BMP-2. Normal C57Bl/6 mice and A2A receptor gene knockout C57Bl/6 mice were selected to make bone defect models. Adenosine A2A receptor agonists and BMP-2 were used locally to observe bone repair. By means of micro-CT, tissue section, and immunohistochemistry, it was found that the bone repair of normal C57Bl/6 mice treated with adenosine A2A receptor agonists was significantly accelerated, and the effect was similar to that of BMP-2. Bone repair in C57Bl/6 mice with A2A receptor gene knockout has been found to be slower than that in normal mice.

Bekisz et al. [38] studied the repair of bone defects with bioactive ceramics that carry adenosine A2A receptor agonists. By measuring the radius of the bone defects, it was found that adenosine A2A receptor agonists could promote bone repair and reduce the area of bone defects. Adenosine A2A receptor agonists can promote fracture healing, but the exact mechanism behind this is unclear.

The adenosine A2A receptor plays a regulatory role in immunity [21, 25, 26] and is expressed on the surface of T cells. The activation of receptors inhibits the proinflammatory effect of T cells and reduces the synthesis and release of proinflammatory factors [21, 26]. Adenosine A2A receptors on T cells affect whether the T cells are immunosuppressed or activated [21]. In the current study, we found that adenosine A2A receptor activation plays a role not only by directly activating A2A receptors on T cell surfaces but also by indirectly regulating T cells through IL-6. Adenosine A2A receptor agonists can change the proportion of the Treg and Th17 cells in blood after fracture, hence reducing the level of IL-6 in the blood and fracture sites. IL-6 can reverse the regulation of adenosine A2A receptor agonists on the proportion of the Treg and Th17 cells in the blood of fracture rats. These results indicate that adenosine A2A receptor agonists regulate the proportion of the Treg and Th17 cells in the blood of rats with fractures and are related to IL-6. Treg cells mainly play an anti-inflammatory role. It has been reported that in rheumatoid diseases, adenosine A2A receptor agonists can reduce rheumatoid inflammation and tissue damage [39]. Boros et al. [40] studied adenosine and ischemia-reperfusion injury. The activation of adenosine A2A receptors can include vasodilation, renin release, and suppression of excessive inflammatory responses of immune cells, as well as mitigation of vascular endothelial cell damage [39, 40]. Not only T cells but also the activation of adenosine A2A receptors can inhibit the proinflammatory response of neutrophils, macrophages, and other lymphocytes. The adenosine A2A receptor agonist can upregulate IL-10 and alleviate myocardial ischemia-reperfusion injury. The level of IL-10 in mice knocked out of the adenosine A2A receptor gene was shown to be significantly lower than that in normal mice. Bortoluzzi et al. [41] studied the lymphocyte of patients with systemic lupus erythematosus and found that the A2A receptor on the surface of lymphocytes was significantly higher than that of healthy people, while the A1, A2B, and A3 receptors were not significantly different from those of healthy people. It is suggested that the adenosine A2A receptor may be an important pathway involved in immune regulation and inflammatory response.

Normal immune function is very important for fracture healing. Impaired immune function or pathological enhancement will affect fracture healing and even cause fracture nonunion. T lymphocytes are important cells involved in adaptive immunity [42–44]. Animal studies have found that the loss of T lymphocytes or a significant reduction in the number of T lymphocytes can affect the maturation of osteoblasts and lead to slow bone mineralization [42–45]. Fracture repair and bone remodeling have been shown to be slow in rats with T lymphocyte deficiency [42–45]. Bone marrow transplantation can reverse the slow healing of fracture in animals with low T lymphocyte immunity [44, 45]. Clinically, it was also found that the bone fragility of HIV patients increased and the rate of osteoporosis was significantly higher than that of normal people [46]. Some studies have found that the rate of fracture nonunion in HIV patients is significantly higher than that in normal people [46, 47]. Jiang et al. [48] reported that the proportion of Treg cells in patients with a nonunion fracture was significantly lower than that in patients with a normal union fracture. Further studies have shown that the Treg cell secretory function was significantly decreased in patients with nonunion fractures. Animal studies and clinical observations have shown that T cells indeed play an important role in fracture nonunion.

IL-6 is one of the most important cytokines regulating T lymphocyte function [24]. T lymphocytes also regulate other cell functions through IL-6. It can be said that there are interactions between IL-6 and T cells in the process of fracture healing. In the early stage of a fracture and local tissue damage, macrophages and lymphocytes in blood collect, and a large number of inflammatory factors are secreted like IL-6. Some studies have found that IL-6 knockout mice have defects in fracture repair, and the rate of fracture nonunion is increased significantly [49, 50]. The injection of IL-6 into the tail vein after fracture in normal mice also has resulted in nonunion fractures [49, 50]. This indicates that IL-6 should be maintained within a certain range after fracture and that it is beneficial for fracture healing.

IL-6 is the most important cytokine for regulating the transformation of CD4+ T cells into Treg cells or Th17 cells [24]. IL-6 promotes the transformation of CD4+ T cells into Th17 cells and the secretion of IL-17 through Th17 cells. At the same time, IL-6 inhibits the transformation of CD4+ T cells into Treg cells. Treg cells and Th17 cells play opposite roles in fracture healing. Th17 cells can recruit and activate other immune cells, secrete a large number of IL-17, act on osteoclast precursor cells, or directly mediate the formation of osteoclasts, resulting in bone resorption. Th17 cells promote osteoclast formation mainly by secreting IL-17. As a proinflammatory mediator, IL-17 can stimulate the expression of IL-6, IL-8, and the colony-stimulating factor (CSF). In the early stage of inflammation, IL-17 can recruit and mobilize a large number of neutrophils into the inflammation area, mediating the infiltration of inflammatory cells and tissue damage. Th17 cells secrete a large number of IL-17, which promotes osteoclast generation, inhibits osteoblast activity, and raises more inflammatory cells, resulting in further destruction of the local fracture tissue and even fracture nonunion in severe cases.

**Figure 10:**
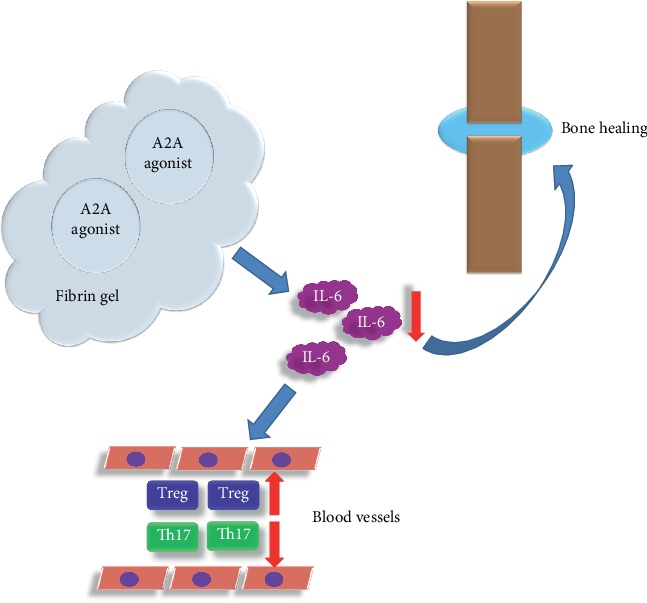
A brief diagram of the mechanism of the adenosine A2A receptor agonist promoting tibial fracture healing in rats. The adenosine A2A receptor agonist enters the tissue through the fibrin gel sustained release system. This reduces IL-6 levels in the blood and local tissues of the fracture, thus promoting fracture healing and regulating the Treg and Th17 cells.

Treg cells and Th17 cells have opposite effects. Treg cells enhance the activity of osteoblasts and inhibit the function of osteoclasts. Treg cells can secrete inhibitory cytokines such as TGF-beta and IL-10 and participate in anti-inflammatory responses [22, 23]. Our results indicate that besides promoting fracture healing, adenosine A2A receptor agonists can also regulate the Treg and Th17 cells in the blood of fracture rats. By injecting IL-6 into the tail vein after making a fracture model, it was found that IL-6 could reverse the effect of the adenosine A2A receptor agonist on fracture healing and the regulation of the Treg and Th17 cells in blood. It is suggested that the promotion of fracture healing through adenosine A2A receptor agonists is closely related to IL-6; it may also be related to the Treg and Th17 cells in the blood. In the future, we will study the relationship between the Treg and Th17 cells in blood and the promotion of fracture healing by adenosine A2A receptor agonists (Figure 10).

## 5. Conclusion

Adenosine A2A receptor agonists can promote tibial shaft fracture healing. At the same time, it can increase Treg cells and decrease Th17 cells in the blood of rats with fracture. The current study also confirmed that the above effects are related to IL-6.

## Data Availability

Please contact the author for data requests.
